# Anti-NMDAR and anti-MOG antibody double-positive encephalitis temporally associated with cytomegalovirus detection in cerebrospinal fluid: a case report

**DOI:** 10.3389/fimmu.2026.1805851

**Published:** 2026-06-09

**Authors:** Rujue Guo, Qili Chen, Lingen Kong, Aizheng Huang, Yankun Li, Caiming Li

**Affiliations:** 1 Department of Neurology, Huizhou First Hospital, Huizhou,Guangdong, China; 2 The First Clinical Medical College, Guangdong Medical University,Zhanjiang, Guangdong, China

**Keywords:** anti-MOG antibodies, anti-NMDAR antibodies, autoimmune encephalitis, cytomegalovirus, MNOS

## Abstract

The co-occurrence of MOG and NMDAR antibodies has been reported in a limited number of cases and is termed the overlapping syndrome (MNOS). Viral coinfections have been identified in a subset of patients with MNOS. Herein, we report the first case of MNOS with concomitant cytomegalovirus (CMV) infection detected in cerebrospinal fluid, a finding that helps to further explore the relationship between viral infection and MNOS. A previously healthy 49-year-old man developed fever and behavioral abnormalities following prodromal symptoms. Metagenomic next-generation sequencing (NGS) of the CSF identified CMV DNA with high confidence (specific reads: 362; relative abundance: 85.97%). Serology was positive for CMV IgG but negative for IgM; serum CMV-DNA detected by real-time PCR was negative. Positivity for anti-NMDAR antibodies and anti-MOG antibodies in the CSF, whereas only anti-MOG antibodies were detected in the serum. The patient’s condition gradually improved after treatment with antiviral agents, corticosteroids, and intravenous immunoglobulin. The main limitations of this report include the lack of detection of CMV-DNA in CSF by real-time PCR, as well as the absence of dynamic assessment of serum/CSF CMV IgG/IgM, anti-NMDAR, and MOG antibody titers. Clinical vigilance for coexisting autoimmune encephalitis should be heightened following viral infections.

## Introduction

Anti-N-methyl-D-aspartate receptor (NMDAR) encephalitis is the most common type of autoimmune encephalitis, accounting for approximately 78–86% of cases (1). Antibodies against myelin oligodendrocyte glycoprotein(MOG)are associated with a spectrum of idiopathic inflammatory demyelinating disorders, collectively termed myelin oligodendrocyte glycoprotein antibody-associated disease (MOGAD). A limited number of cases have reported the coexistence of MOG and NMDAR antibodies, described as overlapping syndrome (MNOS) (2). Additionally, viral coinfections have been identified in a subset of patients with MNOS. Cytomegalovirus (CMV), a member of the Herpesviridae family and also known as human herpesvirus type 5, is a common virus that typically remains asymptomatic in healthy individuals. Following primary infection, CMV establishes lifelong latency within the host and can reactivate upon immunosuppression. This virus has been linked to several neurological disorders, including Parkinson’s disease, Alzheimer’s disease, Huntington’s disease, Autism spectrum disorder, Bell’s palsy, ataxia, and brain tumours, which can cause a wide range of symptoms and challenges for those affected (3). Here, we report the first case of MNOS with concomitant CMV infection detected in cerebrospinal fluid.

## Case presentation

A previously healthy 49-year-old male presented with a 3-day history of nasal congestion and rhinorrhea, followed by fever (37.7 °C) and acute neuropsychiatric symptoms including lethargy, incoherent speech, agitation, shouting, and disrobing over the preceding 24 hours. Emergency cranial CT showed no significant abnormalities, and the patient was admitted for further neurological evaluation. The patient’s family denied any history of chronic diseases such as hypertension, diabetes mellitus, or epilepsy. There was no reported history of food or drug allergies, nor any history of surgery. The patient had no history of smoking or alcohol abuse, and there was no family history of psychiatric disorders. On admission, his vital signs were stable: temperature 36.8 °C, pulse 95 bpm, respiratory rate 20 bpm, blood pressure 139/92 mmHg, and SpO_2_ 99% on room air. No jaundice was observed on the skin, and no palpable lymph nodes were detected. Breath sounds were clear bilaterally, with no dry or moist rales on auscultation. The abdomen was flat and soft, without significant tenderness or rebound tenderness. He was conscious but only partially coherent and uncooperative during examination, with evident impairment of higher cortical functions. Neurological examination revealed bilateral pupils 3 mm in diameter, reactive to light, and normal extraocular movements. Muscle strength was normal (grade 5) in all limbs, and the neck was supple without meningeal signs. The plantar reflex was extensor on the right and flexor on the left. Lumbar puncture demonstrated an elevated opening pressure of 200 mmH_2_O (normal: 80–180) and pleocytosis with a white blood cell count of 60×10^6^/L (normal: 0–8). Cerebrospinal fluid (CSF) biochemistry (glucose, chloride, and protein) was within normal limits. Acid-fast bacilli smear of the CSF showed no acid-fast positive bacteria, and India ink staining revealed no Cryptococcus neoformans. Routine blood tests revealed mild anemia (hemoglobin 126 g/L; normal: 130–175 g/L) and an inflammatory response characterized by elevated neutrophil percentage (75.2%) and C-reactive protein (11.9 mg/L; normal: 0–10 mg/L). Coagulation function tests showed D-dimer 1.74 mg/L (normal: 0–0.55 mg/L) and fibrinogen 4.6 g/L (normal: 2–4 g/L). Serology was positive for cytomegalovirus (CMV) IgG but negative for IgM; serum CMV-DNA detected by real-time PCR was negative. Metagenomic next-generation sequencing (NGS) covering over 20,000 pathogenic microorganisms (including bacteria, fungi, viruses, and parasites) was performed on cerebrospinal fluid CSF, and CMV DNA was detected with high confidence (specific reads: 362; relative abundance: 85.97%). Autoimmune antibodies were tested by a third-party independent medical laboratory (Kingmed Diagnostics, Guangzhou) using the cell-based assays (CBAs) in accordance with the laboratory-developed test standards. The CSF sample was tested for 24 different types of neuronal autoantibodies. The targeted antigens were NMDAR, AMPAR1, AMPAR2, LGI1, CASPR2, GlyR, GABAAR, GABABR, IgLON5, DPPX, DRD2, GAD65, mGluR1, mGluR5, Neurexin-3α, gAChR, KLHL11, GluK2, AK5, AGO, CaVα2δ, AQP4, MOG, and GFAP. While antibodies against MOG were tested using the live CBA method, the other antibodies were tested using the fixed CBA method. The results demonstrated coexisting positivity for anti-NMDAR (titer 1:32) and anti-MOG (titer 1:100) antibodies in the CSF. Subsequently, serum was tested specifically for anti-NMDAR (fixed CBA) and anti-MOG (live CBA) antibodies; only anti-MOG antibodies (titer 1:32) were detected in the serum (**Figure 1**). Thyroid function tests, rheumatoid factor, anti-nuclear antibody, anti-dsDNA antibody, anti-ANCA antibody, HIV antibody, and Treponema pallidum antibody were negative. Brain MRI revealed hyperintense signals on T2-FLAIR and T2-weighted images in the left frontal and temporal cortices (**Figures 2A, B**). Electroencephalography showed diffuse slowing with loss of the posterior dominant rhythm and widespread delta activity. Plain computed tomography of the chest and abdomen revealed no significant abnormalities. On the second day after admission, we initiated ganciclovir (5 mg/kg every 12 hours) combined with high-dose methylprednisolone sodium succinate pulse therapy (1 g/day for 5 days, followed by 0.5 g/day for 3 days, 0.25 g/day for 2 days, and 0.125 g/day for 1 day), sequentially tapered with oral prednisone (initial dose 1 mg/kg/day). On hospital day 4, thrombosis was detected in the intermuscular veins of the right calf. Pulmonary CT angiography ruled out pulmonary embolism, and anticoagulation with dalteparin sodium 5000 IU every 12 hours was initiated. On hospital day 13, the patient showed no clinical improvement, with episodes of excitement and incessant rambling speech alternating with a stuporous state. Therefore, on hospital day 14, intravenous immunoglobulin (IVIG) pulse therapy was initiated at 0.4 g/kg/day for 5 days, followed by olanzapine 5 mg once daily to manage the psychiatric symptoms. On hospital day 25, the patient developed generalized jaundice. Liver function tests revealed total bilirubin 97.7 µmol/L (normal: 0–26 µmol/L), direct bilirubin 18.2 µmol/L (normal: 0–8 µmol/L), alanine aminotransferase 191 U/L (normal: 9–50 U/L), and aspartate aminotransferase 53 U/L (normal: 15–40 U/L). Ganciclovir-induced liver injury was suspected, therefore, ganciclovir was discontinued and hepatoprotective therapy with magnesium isoglycyrrhizinate was initiated. On hospital day 27, the patient’s clinical condition began to improve. He regained clear consciousness and could engage in simple communication. Subsequently, mycophenolate mofetil (0.5 g twice daily) was given as maintenance immunotherapy. The timeline of the patient’s main treatment course is shown in **Figure 3**. On hospital day 30, repeat CSF NGS confirmed negative CMV DNA. Repeat brain MRI showed resolution of the previously noted cortical hyperintensities (**Figures 2C, D**). For economic reasons, the patient declined repeat testing for NMDAR and MOG antibodies in both cerebrospinal fluid and blood. On hospital day 45, his Mini-Mental State Examination (MMSE) score at discharge was 18. At the 60-day follow-up after discharge, the patient exhibited no psychiatric abnormalities, had achieved full independence in daily activities, returned to his previous occupation, and his MMSE score had improved to 25.

**Figure 1:**
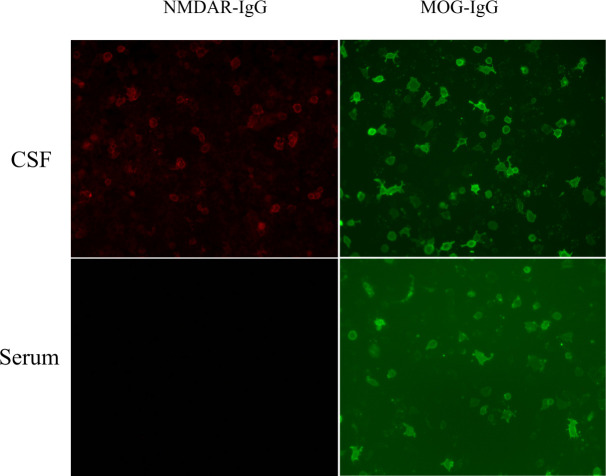
Cell-based assay (CBA) results for serum and CSF antibodies. Anti-NMDAR antibodies were positive in CSF but negative in serum; anti-MOG antibodies were positive in both CSF and serum.

**Figure 2:**
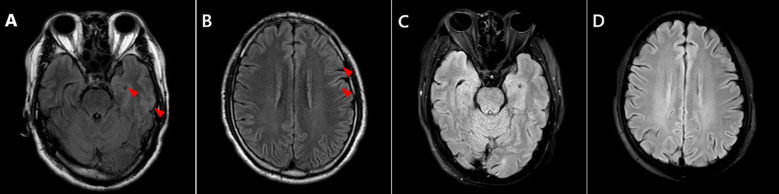
Brain MRI of a patient with MNOS at onset and following treatment. **(A, B)** T2-FLAIR images at disease onset demonstrate focal cortical hyperintensity in the left frontal and temporal lobes (red arrows). **(C, D)** Post-treatment images show resolution of the previously noted cortical hyperintensities.

**Figure 3:**
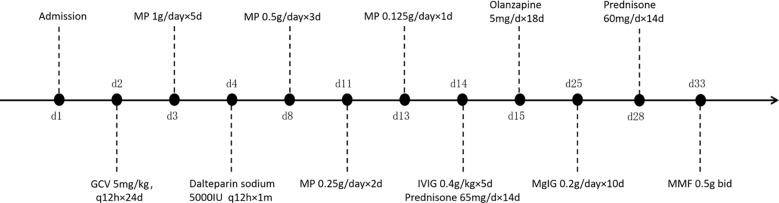
The timeline of the patient’s main treatment course. GCV, ganciclovir; MP, methylprednisolone; IVIG, intravenous Immunoglobulin; MgIG, magnesium isoglycyrrhizinate; MMF, mycophenolate mofetil.

## Discussion

MNOS is a recently proposed concept describing the simultaneous or sequential presence of both anti-NMDAR and anti-MOG antibodies in a patient. Study findings (4, 5) indicate that MNOS predominantly affects young and middle-aged males and is rarely associated with tumors. The clinical presentation of MNOS is primarily characterized by features of anti-NMDAR encephalitis, such as psychiatric symptoms, altered consciousness, sleep disturbances, cognitive impairment, and seizures. However, it can also manifest clinical characteristics typical of MOGAD, including autonomic dysfunction, visual impairment, ataxia, limb paralysis, and numbness. In MNOS patients with positive MR imaging findings, lesions may involve areas such as the cerebral cortex, basal ganglia, cerebellum, and brainstem (2).

Notably, the MNOS case we report presented initially with fever and neuropsychiatric abnormalities. Brain MRI revealed cortical involvement consistent with features often seen in anti-NMDAR encephalitis, without clinical symptoms or MRI findings typically associated with MOGAD. Potential explanations for this presentation are as follows. First, the clinical phenotype of MNOS may be biased toward anti-NMDAR encephalitis, lacking features of MOGAD. Second, the early use of aggressive and effective treatment in this case may have prevented the clinical manifestations associated with anti-MOG antibodies. Currently, cases detecting anti-NMDAR or anti-MOG antibody positivity in viral encephalitis/meningitis are exceedingly rare, with most reports being individual case studies (6–12). Ishimaru et al (6) reported two cases of Varicella-Zoster Virus (VZV)-associated central nervous system infection where anti-MOG antibodies were detected. In one case, anti-MOG antibodies were detected only after clinical deterioration. This patient had a high viral load of VZV detected in the CSF on the first day of admission, at which time anti-MOG antibodies were not present in either serum or CSF. Following clinical worsening, anti-MOG antibodies were subsequently detected in the CSF on day 9 and in the serum on day 11. This confirmed that anti-MOG antibodies emerged after VZV infection, suggesting that viral infection may trigger autoimmune encephalitis. Ma et al (13) reported a series of 23 MNOS cases, among which 9 patients had prodromal infection symptoms. However, no etiological testing was performed to confirm the specific pathogens in those cases.

In this patient, positive serum CMV IgG, negative IgM, and negative serum CMV-DNA detected by real-time PCR suggested a past infection with CMV. The detection of high-confidence specific CMV DNA sequences in CSF by NGS provided evidence supporting the presence of CMV infection in the CSF. Meanwhile, anti-NMDAR and anti-MOG antibodies were concurrently detected in the CSF, with a temporal coincidence between the two, suggesting a possible association between CMV and MNOS. A systematic search of PubMed using the specified keywords revealed no prior reports as of 2025. Therefore, this case represents the first report of temporal coexistence of CMV infection and double-antibody-positive encephalitis.

The replication of the CMV genome is closely associated with nucleotide excision repair (NER)-related factors and the host DNA damage response. It is estimated that CMV encodes approximately 200 proteins, four long non-coding RNAs, and 26 microRNAs (miRNAs), which may interact with human cell biology. However, despite the biologically plausible mechanisms, the association between CMV infection/reactivation and neurological disease is mechanistically complicated. For example, a recent systematic review by Sanami et al. (3) extensively examined the connection between CMV infection/reactivation and a variety of neurological diseases and found that the contribution of CMV to CNS damage can occur via multiple mechanisms, including direct viral injury, immune-mediated mechanisms, neuroinflammation, endothelial dysfunction, and blood-brain barrier breakdown. Nevertheless, it should be noted that in many cases, distinguishing causative infection from coincidental virus detection or immunological abnormalities can be difficult. Thus, we should understand our case as being caused by CMV-associated MNOS and not as an experimental evidence of CMV-induced MNOS. Future studies are needed to explore the association between viral infection and autoantibodies. This case report has several limitations. First, due to limitations of the detection kit, we only tested serum CMV-DNA (by real-time PCR); CSF CMV-DNA (by real-time PCR) was not tested, which could have provided further evidence for central nervous system CMV infection. Second, dynamic assessment of paired serum/CSF CMV IgG and IgM was not performed. Third, dynamic changes in NMDAR and MOG antibody titers in CSF and blood were not monitored.

## Conclusions

In summary, we report the first case of anti-MOG and anti-NMDAR antibody-positive encephalitis occurring synchronously with cytomegalovirus infection. This finding provides new insights into the relationship between viral infection and autoimmune encephalitis, and suggests that patients with viral encephalitis or meningitis require close monitoring for the possible coexistence of autoimmune encephalitis. Conversely, in patients presenting with fever and psychiatric symptoms suggestive of autoimmune encephalitis, attention should also be paid to the possibility of concurrent viral infection, which can be confirmed by CSF PCR or NGS.

### Patient perspective

Despite a tortuous and dangerous treatment course, I greatly appreciate the medical team’s extensive efforts in restoring my health. I have now fully recovered to my pre-morbid condition without any discomfort and remain on low-dose prednisone and mycophenolate mofetil.

## Data Availability

The raw data supporting the conclusions of this article will be made available by the authors, without undue reservation.
